# Rapidly Involuting Congenital Hemangioma of the Hand

**Published:** 2017-03-08

**Authors:** Daniel Delgadillo, Logan W. Carr, Morgan S. Brgoch, John M. Ingraham

**Affiliations:** ^a^Michigan State University College of Human Medicine, Grand Rapids; ^b^Division of Plastic Surgery, Department of Surgery, Pennsylvania State University College of Medicine, Hershey

**Keywords:** congenital hemangioma, vascular malformation, tumor, RICH, NICH

## DESCRIPTION

**Figure 1:**
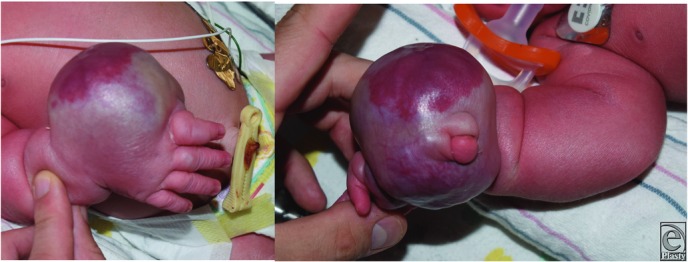
Right hand with rapidly involuting congenital hemangioma.

A 1-day-old male infant was evaluated in the neonatal intensive care unit for a “large vascular mass encircling his right hand” (Fig 1). Ultrasonography, magnetic resonance imaging (MRI), and incisional biopsy were performed, resulting in the diagnosis of a rapidly involuting congenital hemangioma (RICH).

## QUESTIONS

1. **Define congenital hemangioma and the subtypes?**
2. **What are the clinical features and how are they diagnosed?**
3. **What is Kaposiform hemangioendothelioma (KHE)?**
4. **What are the treatment/management options?**

## DISCUSSION

Congenital hemangiomas are rare benign vascular tumors that commonly present fully formed at birth, unlike infantile hemangiomas. The incidence of cutaneous congenital hemangiomas is unknown, and its pathogenesis remains poorly understood. Two congenital hemangioma subtypes have been most recognized: RICHs and noninvoluting congenital hemangiomas (NICHs). Recently, distinction between the two has been called into question, as not all RICHs completely involute and present as NICHs.^1^ Histopathological findings of congenital hemangiomas are distinguished from infantile hemangiomas by the lack of glucose transporter-1 protein (GLUT-1). However, there is very little histological separation between RICHs and NICHs. Histopathologically, they present with varying sized lobules composed of capillary proliferations embedded in a dense fibrous stroma surrounded by large dysplastic vessels.^2^ The deposition of hemosiderin and calcifications are also commonly observed.

**Figure 2:**
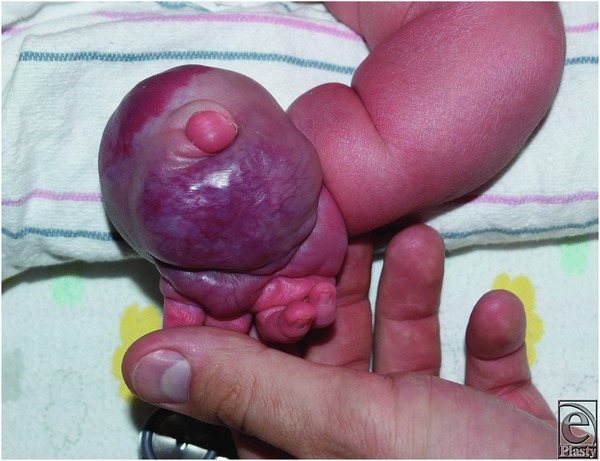
Right hand with a pink to violaceous exophytic mass and coarse telangiectasias.

**Figure 3:**
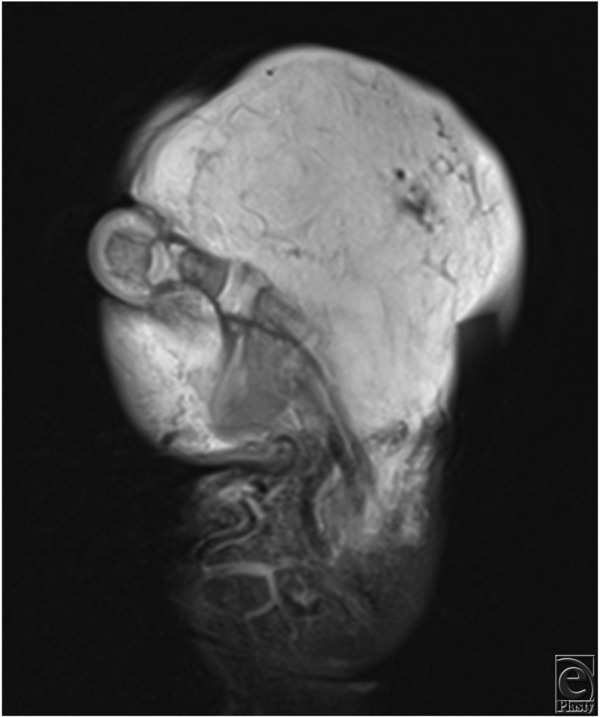
T1-weighted magnetic resonance image of the patient's right hand.

Clinically, both RICHs and NICHs present as solitary, plaques, or exophytic lesions of varying size.^3^ RICHs are distinguished from NICHs by the rapidly involuting process that occurs within the first days to weeks after birth. Furthermore, RICHs typically occur within the head, neck, and lower extremities.^1^ A variety of morphological variants have been described including raised violaceous mass with notable peripheral veins, exophytic vascular tumor with central ulceration, and pink to violaceous exophytic mass with coarse telangiectasias (Fig 2). NICHs present as well-circumscribed, oval, plaque-like masses containing overlying telangiectasias with a rim of pallor. Moreover, 2 morphological variants have been described: patch type characterized by a flat or slightly atrophic surface, and nodular/plaque type associated with a more prominent soft-tissue fullness.^4^ The majority of RICHs completely involute by 14 months, whereas NICHs continue to grow in proportion with the child.^3^ The diagnosis of congenital hemangiomas is made clinically, but imaging such as ultrasonography and MRI (Fig 3) may provide further insight into unclear cases. If suspicion for KHE exists, a biopsy of the lesion should be performed as in this case.

KHE is a rare, locally aggressive vascular tumor that often presents in early childhood and difficult to distinguish from a congenital hemangioma. It is often found in conjunction with the coagulopathy disorder Kasabach-Merritt syndrome. The pathogenesis of KHE is poorly understood, but spindled neoplastic endothelial cells demonstrate expression of vascular markers CD31 and CD34, lymphangiogenesis markers vascular endothelial growth factor receptor (VEGFR-3), and lymphatic markers D2-40 and PROX1.^5^ Clinically, KHE is described as an indurated subcutaneous mass with a bruised appearance and occasional telangiectasias. KHE most commonly occurs on the extremities or trunk and is diagnosed through biopsy illustrating lobules of closely packed spindle cells infiltrating the dermis, subcutaneous fat, and muscle.^6^

Management of congenital hemangiomas is patient specific and highly dependent on the size, location, ability to involute, and presence of complications. RICHs are self-resolving and usually expectantly managed, unless complications such as ulceration or bleeding occur. Once involution is complete, the fibrofatty residual tissue may be excised to enhance cosmesis. Contrary to RICHs, NICHs do not resolve over time but can be managed similarly if the patient is asymptomatic.^7^ Surgical management is generally reserved for patients who present with symptoms, as the tumor continues to grow in proportion with the child. As for KHE, wide local excision is the current treatment of choice with the option of adjunctive chemotherapy such as vincristine, actinomycin D, and cyclophosphamide.
